# Relations of power and oppression within the delivery room: nursing narratives

**DOI:** 10.1590/1980-220X-REEUSP-2021-0476en

**Published:** 2022-05-20

**Authors:** Yesica Yolanda Rangel-Flores, Consuelo Magdalena Martínez-Villa, Vanesa Jiménez-Arroyo

**Affiliations:** 1 Universidad Autónoma de San Luis Potosí, Facultad de Enfermería y Nutrición, San Luis Potosí, México.; 2 Universidad Autónoma de Chihuahua, Facultad de Enfermería y Nutriología, Chihuahua, Chih, México.; 3 Universidad Michoacana de San Nicolás de Hidalgo, Facultad de Enfermería, Morelia, Mich, México.

**Keywords:** Violence, Gender-Based Violence, Workplace violence, Nursing, Delivery rooms, Qualitative research, Feminism, Violência, Violência de Gênero, Violência no trabalho, Enfermagem, Salas de parto, Pesquisa qualitativa, Feminismo, Violencia, Violencia de género, Violencia laboral, Enfermería, Salas de parto, Investigación cualitativa, Feminismo

## Abstract

**Objective:**

To analyze, from the perspective of decolonial feminism, the power and oppression relations experienced by nurses in the delivery room in a hospital in Mexico.

**Method:**

Qualitative study in which 15 nurses selected by theoretical sampling were interviewed. The interviews were fully transcribed and subsequently analyzed using content analysis.

**Results:**

The emerging central category was “Inter- and intragender power/oppression relations” and psychological and symbolic violence were the most frequent types. Gender was confirmed as the most important structural determinant of oppression, cutting across bodies and professional identities. The conditions contributing to intragender conflict are age, expertise, and specialization. Three coping resources were documented: defenselessness, complicity and resistance.

**Conclusion:**

It is necessary to denaturalize the forms of power/oppression sustained by gender inequalities, but also to discuss other conditions that determine power/oppression relations between women and colleagues. Eradicating intra-gender and intergender violence is necessary to access safe working environments that promote creativity for the exercise of care.

## RESUMEN

**Objetivo:**

Analizar desde la perspectiva del feminismo descolonial, las relaciones de poder y opresión que viven enfermeras dentro de la sala de parto en un hospital de México.

**Método:**

Estudio cualitativo en el que se entrevistó a 15 enfermeras seleccionadas por muestreo teórico. Las entrevistas transcritas en totalidad fueron sometidas posteriormente a análisis de contenido.

**Resultados:**

La categoría central emergente fue “Relaciones de poder/opresión inter e intragénericas” y las formas más frecuentes de violencia fueron psicológicas y simbólicas. El género se confirmó como el condicionante estructural más importante de la opresión, atravesando los cuerpos y las identidades profesionales. Las condiciones que contribuyen al conflicto intragénero son la edad, la expertis y la especialización. Se documentaron tres recursos para el afrontamiento: indefensión, complicidad y resistencia.

**Conclusión:**

Es necesario desnaturalizar las formas de poder/opresión sustentadas en las desigualdades de género, pero también discutir otras condiciones que determinan relaciones de poder/opresión entre mujeres y colegas. Erradicar las violencias intragenéricas e integénericas es necesario para acceder a entornos laborales seguros y que potencien la creatividad para ejercer el cuidado.

## RESUMO

**Objetivo:**

Analisar as relações de poder e opressão vivenciadas por enfermeiras na sala de parto de um hospital no México sob a perspectiva do feminismo decolonial.

**Método:**

Estudo qualitativo no qual foram entrevistadas 15 enfermeiras selecionadas por amostragem teórica. As entrevistas transcritas na íntegra foram posteriormente submetidas à análise de conteúdo.

**Resultados:**

A categoria central emergente foi “Relações inter e intragênero de poder/opressão” e as formas psicológica e simbólica de violência foram as mais frequentes. O gênero foi o mais importante determinante estrutural da opressão, atravessando corpos e identidades profissionais. As condições que contribuem para o conflito intragênero são idade, perícia habilidade e especialização. Três recursos de enfrentamento foram documentados: vulnerabilidade, cumplicidade e resistência.

**Conclusão:**

É necessário desnaturalizar as formas de poder/opressão sustentadas pelas desigualdades de gênero e discutir outras condições que determinam as relações de poder/opressão entre mulheres e colegas. Erradicar a violência intragênero e intergênero é necessário para acessar ambientes de trabalho seguros que promovam a criatividade para o exercício do cuidado.

## INTRODUCTION

Even though violence has been one of strongest lines of research in the last two decades, given the complexity of the phenomenon, it is necessary to delve into the subject and incorporate emerging theories in the field of gender and human rights into its analysis, as well as methodological and epistemic resources that help to generate new explanations for the motivations of violence and the coping strategies implemented against it^(1)^.

On the other hand, it is urgent to study the violence that takes place in spaces other than the domestic and, like the so-called “family violence”, reduces the quality of life and health. Revealing and explaining the violence that happens in the workplace and specifically within the hospital setting is particularly relevant. In this place, power/oppression relations legitimize forms of exploitation in which nurses, due to their double or triple condition, gender and profession, –sometimes ethnicity and class–, appear as particularly vulnerable^(2)^.

Despite the fact that several studies speak about the importance of nurses’ role in promoting a culture of peace, particularly because of their responsibility during screening for the early identification of domestic violence and referring victims to specialized services, including referrals to complaint and refuge centers^(3)^. Little has been written about the violence experienced by nurses within health services, and about its motivations and impacts on nurses’ personal and professional development^(4)^.

Nonetheless, previous studies make it possible to realize that the violence faced by nurses in the workplace is closely associated with the feminization of the profession^(2–5)^. Some works addressing the problem of violence within health services demonstrate that compared to other health professionals, nursing personnel are more vulnerable to experience bullying and harassment by superiors and members of the multidisciplinary team^(6,7)^, particularly the younger and less experienced professionals^(8)^. As for the identity of aggressors, both service users^(9)^ and colleagues from the healthcare team^(10)^, particularly doctors, are included^(11)^. Regarding the forms of violence, an integrative review documented that moral harassment is the prevailing form^(7)^.

In the context described above, one of the limitations identified in these studies is that the analysis of violence within the hospital setting is limited to parameters established for workplace violence, mainly within a perspective of administrative matters and organizational type of health institutions^(12,13)^, without delving into how a series of structural issues such as gender, class and profession trigger and guide the dynamics of the conflict. Little is written about the impacts generated by socially constructed inequality between genders within the health field, or the stereotyping/gendering of professions, a phenomenon that makes invisible and even naturalizes the oppressions exerted by masculinized professions on feminized professions.

In this study, we used the contributions of decolonial feminism to observe and interpret the phenomenon. Decolonial feminism emerges as a critical look at hegemonic classical feminism, challenging it to question other categories added to the gender condition that place women in a subordinate position in relation to men. However, unlike classical feminist hegemonic thought, it recognizes the existence of other categories such as class, ethnicity, education, etc., which contribute to the unequal distribution of power, not only of women in relation to men, but also within the gender itself, i.e., among women^(14)^.

Taking this theoretical proposal as a framework allows us to explain how gender constructs permeate professional spaces and translate into resources to stereotype (assign stereotypes) and genderize (assign a gender) professions. This situation has an unfavorable impact on both those who exercise Nursing and on the profession itself, a serious situation, given the evidence that feminized professionals are characterized as being at greater psychosocial risk and having a greater salary gap compared to other professions^(7,15)^.

Likewise, this shows us other structural conditions sustaining the power and oppression relations within health services, since these do not emerge exclusively associated with the sex-gender condition, but with its intersection with other conditions that reinforce inequalities and legitimize oppressions, namely class, ethnic origin and educational level; and also make visible the complicity of institutions to sustain these forms of oppression and inequality^(16)^. In accordance with the foregoing, the objective of this study was to analyze the relations of power and professional oppression experienced by a group of nurses within the delivery room in a public hospital in the north of Mexico from the perspective of decolonial feminism.

## METHODS

### Design of Study

Qualitative study focused on institutional ethnography within the context of the delivery room. Ethnography has a holistic character because it deepens the discourse and enables its triangulation with practices. Thus, it has the potential to transcend even the imaginaries that motivate the actions and interactions of people within a specific field^(17)^.

### Scenario

Public urban hospital in which care is provided to the population through “*Seguro Popular*” (in English, Popular Insurance), a public program that subsidizes a package of free medical services for the population that does not have social security and is at high risk of impoverishment due to health expenses. The decision to work inside delivery rooms was taken because previous studies have mentioned this space as particularly stressful for health personnel hence, a trigger for conflicts and at higher the risk of practicing and suffering some type of violence^(11)^.

### Selection Criteria

Participants were nurses who work in delivery rooms. The number of participants was defined according to theoretical data saturation and selection was according to criteria of theoretical relevance. At first, following the propositional sampling, the attempt was to identify participants with knowledge of the phenomenon, which was assessed through informal conversations held within services as part of the ethnographic experience and before the interviews. In a second moment, the actors who allowed us to deepen the findings identified as the most relevant to theorize were invited to participate. As much actors as needed were asked to participate up to the point of theoretical data saturation, that is, until the shared information began to be redundant and the analysis categories were sufficiently consolidated.

### Methods and Procedures for Data Collection

The data collection period was 2017–2019, when individual interviews were conducted using a semi-structured script based on the theoretical construct of the five radical ideas that support symbolic interactionism, which are: a) societies or human groups, b) social interaction, c) the human being as an agent, d) human acts, e) the interconnection of lines of action^(18)^.

**Table:** 

Dimension	Description
Societies or human groups	Professional identity, belonging to the professional group, identity traits, professional identity conflicts.
Social interaction	Professional relationships and conflicts within the delivery rooms, communication channels and strategies, conflict resolution and work agreement.
The human being as an agent	Acting to resolve professional conflicts and for delimitation of professional functions, limits and connections of professional action with other members of the team.
Human acts	Motivations and personal and/or collective motivations for professional practice, security and confidence in oneself for acting professionally.
The interconnection of lines of action	Agency to act professionally in relations with all other professions.

The ethnography was carried out by the second author of this article, who stayed intermittently in the delivery rooms for a six-month period, indirectly integrating herself in the field, participating in nursing care and taking ethnographic notes. From this first approach, the purpose of the study was informed and the informed consent form was handed for signature. At the request of nurses themselves, the meetings were held outside the institutions where they worked. Conducting the interviews in the library of the teaching hospital and in cafes near the hospital helped them feel free to express themselves and generate deep narratives. Interviews were always conducted by the same researcher and for three participants, more than one meeting was required. Each interview lasted one hour, on average.

### Data Analysis

Data were analyzed immediately after each interview according to the thematic analysis proposal. Subsequently after each meeting, the audios were transcribed, starting the open coding to identify the live codes and propose the emerging categories and subcategories. After this first analysis, it was decided if another meeting was necessary to consolidate it.

### Ethical Aspects

The study was reviewed and approved by the Research Ethics Committee of the Universidad de Guanajuato, registry CIDSC 2950127 and by the Research Ethics Committee of the hospital where this study took place, where it was recorded with registry number 000176 of the book registration of research protocols, volume II.

## RESULTS

We worked with fifteen nurses, the average age was 32.4, with a minimum of 25 and a maximum of 52, all mixed race. Regarding educational level, three were nursing assistants, four were nursing technicians, four were licensed nurses, two had a master’s degree in Nursing, one had a master’s degree in Quality and another a master’s degree in Human Resources. The time of experience in the delivery room area was between two and 16 years.

The central category that emerged was “Inter- and intra- gender power/oppression relations” since it was identified that these relationships are experienced and faced in a significantly different way between men/women and among women.

The predominating forms of violence were psychological and symbolic; psychological violence is exercised mainly from the practice of ‘silent treatment’ or invisibility: *The doctor does not look at your face, he arrives and does not speak to you, does not greet you, he does not say anything* (N6). Although also through words: *Even the doctor, sometimes they come tired or reluctant, any detail, that is, it is not a conflict, but sometimes any word, a tiny detail like that makes them explode and yell for nothing* (N3).

Symbolic violence is not fully identified as an act of oppression or violence, but as a condition that determines a differentiated workload between professions.

[Doctors] *come more to see what they get* [referring to flirting with female doctors, students, nurses], *rather than to really provide care, and that is a problem, because they are just gossiping and do not do what are supposed to do* (…) *Nursing ends up doing it and for this reason, we stop doing what we have to do* (N4).

### Intergender Power/Oppression

The social constructs of gender have the potential to stereotype individual bodies and experiences, but they also do the same with professions. The masculinized professions subordinate the feminized ones, thus reproducing power/oppression relations and dynamics that are very similar to those occurring in the domestic sphere.

*The role of the doctor is to arrive and ask how the lady has been and well, we tell him, and we are the ones who provide more support, right? We are the ones who are here, we know what she has, why she is crying (…) and the doctor, well, no (…) he just gets to what it is, they don’t have that much of a relationship with the patient* (N4).

The incidence of gender in the professional field stimulates a series of behaviors that translate into a greater workload for Nursing. Masculinity seems to be incompatible with performing a series of tasks institutionally associated with medicine so these subjects withdraw themselves, and expect nursing to perform the tasks, even though they are not described as institutional nursing functions and they have time to perform those that they should carry out independently.

*In each area, medical recommendations are followed, while others are not their indications, they are things we know we have to do, something like implicit (…) but it turns out that we are the ones who monitor the infusions and we are on the lookout for foci* [fetal cardiac]*, because sometimes the doctors are there and sometimes they are not, or they are asleep, and you wake them up… And why, what do you want?!… Then save yourself the trouble, do it too* (N7).

*One understands that they know how to do their job and Nursing to do theirs, the problem is they don’t see it that way and they want to tell you how to do your job, although sometimes Nursing ends up doing theirs, they make us do screening that raises medical data and not nursing data, we value what is ours, which will help us to provide better care, even if the NCP* [Nursing Care Process] *is not done, but that’s how it begins, but no, for them, Nursing is not important, because they don’t they know it and they come to make us to do their thing* (N13).

### Intragender Power/Oppression

The dispute in power/oppression relations within the institutional space was not only documented in the doctor-nurse relationship, but also evidenced within the profession itself and among women. Nurses who achieve higher levels of specialization replicate these power/oppression relations with younger nurses, those with less expertise and those without specialization because they do not have the resources to do so or simply because this is not of their interest.

*But that they* [the other nurses] *also know how to value someone, one knows more from experience, the piece of paper is indeed necessary, but they humiliate, and one is not here for that* (N6).

*Well* [the more educated nurses] *want to make you feel less, that because you don’t study, you need to read, they believe what they say is the law, and they have the bosses in their pockets and you are the stupid one, it annoys me that they tell me whether to study or not, everyone has their priorities and needs* (N14).

Specialization is not perceived as a condition that enhances the quality of care, but as a determinant that fragments relationships, promotes competition and rivalry among the team, and affects communication and empowerment between peers.

*In our time, if someone of higher rank told you something, you would do it without saying a word and now you say something to someone of less seniority, perhaps more studied, yeah right that they will listen to you! It seems you’re telling them they know nothing and they get upset* (N8).

*Well if they are not good co-workers, they are not good nurses, even if they know a lot because they have studied* (N15).

Regarding the theme of resources used by Nursing to move within these relations of power and oppression, three main strategies were identified: defenselessness, complicity and resistance.

### Defenselessness

Some participants said they had witnessed situations where their colleagues suffered violence without offering resistance. They also conceive that this acceptance of oppression derives from seeing Nursing as a subordinate profession, or because some advantage is identified when tolerating these behaviors.

*The nurse is used to and trained to follow medical indications, sometimes without assessing if it is the right thing to do or not, then, these become routine and they are proud of it, because they already know that such a doctor “likes it” and thus they keep him happy, or yet, he brings them something to eat* (E10).

Another issue demonstrated in this study is that defenselessness emerges as the only possibility within a context that is known to go unpunished, in which justice will not be made. This lack of credibility in the promotion of justice emerges from previous experiences in which more credibility and protection were given to medical personnel than to nursing personnel. Or even for witnessing the re-victimization experienced by nurses when confidentiality is not guaranteed during a complaint or claim process.

*Their attention is called and the doctor gets angry with you and it’s a very tense situation, you better not say anything anyway, the next day they’ll do the same thing, so who do you complain to, where you do find support, or who cares, we shut up and continue, we get to work and try to do it the best way* (N4).

Experiences like these end up reinforcing the collective thought of the subordinate image of Nursing, also affecting the capacity of professionals acting in defense of their patients’ rights.

*As a nurse, maybe I’m just a mortal, maybe I don’t have all your experience, I have my studies, but I don’t dare* (N15).

*No matter how hard I try not to take it personally it’s not possible, sometimes if I get involved* [when she witnesses user mistreatment] *and if it is – don’t yell anymore, don’t yell at him anymore*- [she tells the doctor] *but* [they take it] *the wrong way, that is, they also get upset with someone* (N8).

### Complicity

Living for a long time in a context of violence, regardless of whether it is institutional, entails the risk of normalizing these unacceptable ways of relating and even legitimizing them as valid. In this sense, we identify that complicity is exercised in different ways. For example, ignoring the violence suffered because they find it embarrassing to be mistreated in front of users.

[Doctors] *don’t know how to handle themselves and they talk harshly to a person in front of patients, so you are trying to gain their confidence* [the patient’s], *and then, the patient says, what happened? If he is yelling at you, then what should she expect for? And you try to calm her down and tell her no, it’s not that, it’s another thing, you try to get over the moment* (N4).

Other motivations for not confronting violent attitudes are related to the normalization of this way of relating and the acceptance that within this context, sometimes one acts a victim and at others, as a victimizer.

*Because you are a witness to mistreatment, rude in their treatment, but no way we put them on the cross, after a while it’ll be me and who will defend me, hahaha (…) that’s why anything that can go wrong will go wrong (…)* (N7).

Some participants acknowledge that committing violence within the service is naturalized. Even when it is inflicted against users, they do not identify it as violence but rather as part of the institutional dynamic.

*They* [patients] *don’t even realize they are being mistreated, because they’ve always been treated that way. And who sees us? Nobody! We cover for each other and avoid problems* (N11).

*We are closed services, many things are overlooked here, when we enter, we know it is like that and that’s how we all treat each other, doctors, nurses, cleaning and students, that’s why patients do not notice the difference in treatment, because we all treat each other like that* [violently] (N7).

Developing complicity even starts to be seen as a behavior of solidarity.

*I feel that I am ventilating my own practice, I know these are things that can be improved, but we let ourselves get carried away by what is easy, what has always been done, little by little, the students start to do the same, it has happened to all of us* (N5).

### Resistance

Some people do not align with these dynamics of defenselessness and complicity, and question them. Dialogue stands out as one of the strategies, even though they recognize that trying to dialogue can also be interpreted as a confrontational act.

*With the co-workers, when they shout, I don’t like it at all, if we can solve it by talking, they think shouting will solve it, but I think we can talk and solve everything, but it’s not easy for them to want to talk without yelling* (N5).

*If you confront them you make enemies out of everyone, if you remain silent you get frustrated, what I do is to be different and try to get the others to see me, well I don’t know, the least they might do is what I do one day, I know they won’t like what I’ll tell them and there we go again, I am the troublemaker, the spoiled milk* (N6).

They point out that it is undesirable to involve superiors. In fact, the complaint is seen as a form of violence, so they try to avoid it and resolve the situation by their own means.

*Some people talk to the supervisor straightaway, and others don’t, we try to talk, calm down (…) look, you did this (…) and just like that (…) well, people are different, it depends on who experienced the situation, they yell and so on…then I get scared, but I don’t get the supervisors involved* (N7).

A relevant issue was to realize that dialogue is identified as a possible strategy between nursing professionals, but not when it comes to a situation between a doctor and a nurse. Then, there is a double standard for agreement; gender and profession, two oppressive and determining conditions for not accessing justice.

*You confront them* [doctors] *and it’s very exhausting, they’re very rude, they think they know everything and well, relax, everyone here knows what they have to do, they know, we know* (N8).

*Notice that the freelancers* [doctors] *don’t do that* [be violent]*, the residents and interns do it more, but it’s easier for them if you tell them off, even if they get back at you, but it’s easier than talking to them* (N9).

Nurses know in advance that they are at a disadvantage compared to doctors and prefer confrontation rather than reporting the situation.

*And you tell them off, they complain that you are mistreating them and you know that instead of calling their attention, they call yours, and you choose not to get into trouble anymore and not tell the bosses, you tell them, you tell them off and you know where they are, even if they become rude* (N3).

## DISCUSSION

The findings show that violence within this space is based on power/oppression relations that deserve to be discussed, distinguishing between intragender (between the two genders) and intergender (within the same gender) violence, since the experiences and coping mechanisms are substantially different between men-women and within the same group of women^(19)^.

Regarding power and oppression relations between genders, the existence of symbolic violence stands out. This type is complex to be recognized by participants precisely because as pointed by Bourdieu, it becomes invisible in the eyes of victims and even of aggressors, given the strategies of complicity developed by the State, society and institutions to make these forms of power/oppression legitimate and apparently “natural” in institutional settings, even causing the oppressed to naturalize and justify their oppression. In Bourdieu’s terms, the dominated introject mental dispositions that legitimize power/oppression relations and naturalize them^(20)^.

Symbolic violence was implicit in an unequal distribution of work that is not followed according to the job description but a tendency of the medical staff to offload on nurses the functions of their professional responsibility. The foregoing from gender theory is associated with the phenomenon of gendering of professions, that is, the tendency to classify professions in collective thought as “masculine” and “feminine”. This act reproduces the roles played in the domestic sphere, where routine tasks that require more permanence and are seen as less complex, are deemed feminine, being precisely the activities that contribute directly to greater exhaustion and mental burden^(21)^.

Although participants of this study reported disagreement with the fact of having to assume tasks that correspond to medical personnel, it became evident how they naturalize this overload, which is product of the assimilation of a series of imaginaries built around the professions, from those that legitimize the praxis of what has been called the “authoritarian medical habitus”^(11)^. This concept refers to the symbolic and material construction of institutional interactions based on professional hierarchies that “legitimize” medical personnel to commit acts that contribute to inequality, punishment, discrimination and violation of human rights; both in the relationships maintained with users of services and with the rest of the multidisciplinary team.

The findings reported in this study demonstrate that the processes of coloniality in the health field are not limited to the historical moment of the emergence of Nursing nor are an overcome issue. The power/oppression relations between professions continue to be reproduced, derived from a patriarchal system that is becoming more sophisticated through increasingly subtle strategies. Reinventing oneself and maintaining gender inequalities, both in the domestic sphere and in the public sphere through forms of interaction and mistreatment based on sexism within a work space that is not gender neutral^(22)^ and where the unequal distribution of work is sustained in a colonization of being and power, and also of knowledge^(23)^, and where the humanism sustaining Nursing is conceived as subordinate to the hegemonic biomedical model that sustains medicine^(24)^.

With the foregoing, we can also realize that the devices of power and oppression not only cross sexualized and gendered bodies as men and women, but also do so with the subjectivities sustaining professional action, which leads nurses to embody a double condition of oppression. First as women, and then as professionals in a career considered as subordinate by its feminized character. This largely explains the resource of defenselessness used by nurses in conflict situations with the medical team. This is not a natural resource, but socially and institutionally learned^(20)^, where the lack of access to justice, when violence is reported, successfully installs in nurses’ imagination the idea of defenselessness in the face of impunity to which they are subjected or have seen other colleagues be subjected to.

Defenselessness appears as an individual threat that reduces people’s motivation to think and act to help themselves in a situation identified as adverse. Its scope is highly dangerous for mental health, since it has been associated with a tendency to develop depressive disorders and exerts serious impact on the self-esteem and the sense of “being capable”^(25)^. In addition, defenselessness can generate collective impacts on the profession, repeatedly putting it in a subordinate position and reducing opportunities to transform this imaginary. Younger nurses witness that more experienced nurses tolerate oppression, which translates into the naturalization of gender inequalities within the professional field, an inequality based on gender stereotypes.

Even though intragender relations of power/oppression, as well as those within the team are influenced by gender, they are also affected by other determinants such as age, class and education. This makes it more than clear that indeed, as decolonial feminisms postulate, the gender condition is not the only one generating inequality, and other determinants coexisting within the same group of women must be considered, among which we mention race, nationality, subalternity and social class^(26)^.

This study shows, as already mentioned by other authors, that the youngest and most inexperienced nurses experience the greatest oppression within the hospital setting^(8)^. Similarly, we identified that colleagues who do not undertake specialization studies are seen as less committed to the profession and vocation. Although we did not identify studies conducted specifically with the Nursing population, other studies have indicated that the poorest women with greater limitations for doing specialization courses are more vulnerable to harassment and discrimination both by their peers and within the labor market^(27)^.

It is necessary to analyze the perverse relationship between patriarchy and capitalism, and how this conjugation leads women to compete within the team itself, ignoring and even denying the existence of a series of disadvantages associated with the female gender in the labor market. Ignoring it involves women in disputes that reinforce these multiple disadvantages, and generate inequalities that divide them and thereby reinforce a current social order that continues to harm females as a gender^(28)^. The patriarchal system filters into women’s training as citizens and professionals. From a pedagogy based on cruelty, it enables women to reproduce and care for the interests of a social order based on hierarchical social patterns that classify and divide them based on a series of structural conditions such as gender, ethnicity, class, education, etc^(29)^.

From the foregoing, we realize that the resource of complicity makes sense, although not for nurses or the team, but for a capitalist, neoliberal and patriarchal social order. Power comprises an objective and a subjective character. In its objective character, power is exercised independently of personal will, supported by culture and established in institutions. In its subjective nature, it depends on the operation of a series of thought dispositions introjected by people during socialization processes with the purpose of naturalizing and accepting the hierarchies of oppression. The conjunction of both conditions enables the permanence and apparent immutability of power/oppression relations^(29)^.

As the phrase “whenever domination-exploitation relationships exist, there is resistance, there is struggle”^(29)^, points out, the resistances that we have documented in this study show how these relations of power/oppression are visible to a group of participants. This is extremely satisfying, as it reveals that new thought arrangements are beginning to establish. Furthermore, these arrangements also allow them to propose strategies to change the meaning of these relationships, which translates into a process of personal and professional emancipation, and represents the possibility of developing a counter-hegemonic response to the prevailing colonial and patriarchal view in health institutions. Moving the imaginaries that support the coloniality of being and doing implies the possibility of politicizing the relationships shaping the action within health institutions in a different way, thinking of new forms of solidarity as an action aimed at overcoming injustice-inequality, considering the situation of the other an unfair condition and acting to transform that reality^(30)^.

Finally, it would be of great help to incorporate the proposal of “counter-pedagogies of power” in our professional actions, relearning to relate not only with the masculine gender, but also and above all with women. Finding new ways of thinking and acting collectively, as well as promoting decolonial configurations through which we can rebuild ourselves as a more receptive and creative society, with violence as an increasingly less common practice^(29)^.

## CONCLUSION

The objective of this study was to analyze, from the perspective of decolonial feminism, the relationships of power and professional oppression experienced by nurses in the delivery room in a public hospital in northern Mexico. The findings show the differentiated existence of inter- and intragender power/oppression relations that translate mainly into psychological and symbolic forms of violence. Gender was identified as the most important structural determinant of oppression, crossing bodies and professional identities, while the conditions contributing to intragender conflict are age, expertise and specialization. Three coping resources were documented: defenselessness, complicity and resistance. The existence of resistance strategies represents a temporary opportunity to intervene and promote emancipation. It is necessary to take into account that given the participants’ characteristics, some intersectional categories such as race could not be discussed in depth. In addition, there were few elements to discuss the issue of social class.

It is desirable to promote strategies from higher education institutions, universities and unions that promote collective and team monitoring to encourage the personal and professional emancipation of colleagues within institutional spaces.

Universities must be updated not only in terms of content. It is also necessary to propose a new epistemic and political position of future graduates in society, promote critical thinking paradigms that enable professional autonomy and the development of agency to manage and access violence-free work environments that do not reproduce gender inequalities from the sexist imaginary in wages or workload.

It is also necessary to question the colonial knowledge that we continue to reproduce within educational institutions and learn to control the irrepressible desire to prioritize one type of knowledge over another. In particular, to question how certain types of knowledge distance us from the humanism that should permeate professional action.

## ASSOCIATE EDITOR

Rosa Maria Godoy Serpa da Fonseca
